# Scaffold Protein Lnx1 Stabilizes EphB Receptor Kinases for Synaptogenesis

**DOI:** 10.3389/fnmol.2022.861873

**Published:** 2022-04-21

**Authors:** Na Li, Si Chen, Nan-Jie Xu, Suya Sun, Jin-Jin Chen, Xian-Dong Liu

**Affiliations:** ^1^Research Center of Translational Medicine, Shanghai Children's Hospital, Shanghai Jiao Tong University School of Medicine, Shanghai, China; ^2^Department of Anatomy and Physiology, Shanghai Jiao Tong University School of Medicine, Shanghai, China; ^3^Shanghai Collaborative Innovation Center for Translational Medicine, Shanghai Jiao Tong University School of Medicine, Shanghai, China; ^4^Shanghai Key Laboratory of Reproductive Medicine, Shanghai Jiao Tong University School of Medicine, Shanghai, China; ^5^Key Laboratory of Cell Differentiation and Apoptosis of Chinese Ministry of Education, Shanghai Jiao Tong University School of Medicine, Shanghai, China; ^6^Department of Neurology and Institute of Neurology, Rui Jin Hospital, Shanghai Jiao Tong University School of Medicine, Shanghai, China

**Keywords:** LNX1, EphB receptor, spine, synapse, hippocampal CA3

## Abstract

Postsynaptic structure assembly and remodeling are crucial for functional synapse formation during the establishment of neural circuits. However, how the specific scaffold proteins regulate this process during the development of the postnatal period is poorly understood. In this study, we find that the deficiency of ligand of Numb protein X 1 (Lnx1) leads to abnormal development of dendritic spines to impair functional synaptic formation. We further demonstrate that loss of Lnx1 promotes the internalization of EphB receptors from the cell surface. Constitutively active EphB2 intracellular signaling rescues synaptogenesis in *Lnx1* mutant mice. Our data thus reveal a molecular mechanism whereby the Lnx1-EphB complex controls postsynaptic structure for synapse maturation during the adolescent period.

## Introduction

Synapse formation and stabilization are fundamental to the remarkable specificity of neuronal wiring for learning, memory, and cognition (Lamprecht and LeDoux, 2004; McAllister, 2007; Holtmaat and Svoboda, 2009; Kolodkin and Tessier-Lavigne, 2011). Conversely, improper formation or function of these synapses may lead to neurodevelopmental abnormalities and mental disorders, such as Autism spectrum disorder and schizophrenia (Mirnics et al., 2001; Stephan et al., 2009; van Spronsen and Hoogenraad, 2010; Bourgeron, 2015). Numerous studies over the past decades have demonstrated that functional synapse formation requires coordination of intrinsic genetic programming and extrinsic regulatory factors during postnatal neural development (Scheiffele, 2003; McAllister, 2007; Giagtzoglou et al., 2009; Shen and Scheiffele, 2010; Sheng and Kim, 2011; Siddiqui and Craig, 2011; Sala and Segal, 2014). Recent studies have revealed that glutamatergic neurotransmission is not necessary for the assembly of excitatory synapses in the developing forebrain, which highlights an instructive role for intrinsic mechanism in synapse formation (Lu et al., 2013; Sando et al., 2017; Sigler et al., 2017).

Synapse assembly and maturation are orchestrated by secreted, transmembrane, and intracellular proteins that guide axons to their targets, mediate initial connections, and recruit organizing molecules to active zones and opposed postsynaptic sites (Cohen-Cory, 2002; Tada and Sheng, 2006; Shen and Scheiffele, 2010; Kolodkin and Tessier-Lavigne, 2011; Krueger et al., 2012; Sudhof, 2012). Scaffold proteins containing PDZ domains play critical roles in this process to ensure synaptic efficiency and fidelity (Garner et al., 2000, 2002; Sheng and Sala, 2001; Feng and Zhang, 2009; Williams et al., 2010; Emes and Grant, 2012). Typically, PDZ-domain-containing proteins are able to bind with short peptide motifs of other proteins that are targeted to a restrictive subcellular site of functional receptors or channels to perform a specific function (Garner et al., 2000; Nourry et al., 2003; Kim and Sheng, 2004; Feng and Zhang, 2009). Over the past decades, a large number of PDZ proteins have been identified to participate in numerous biological processes that include synaptic transmission and plasticity (Beique and Andrade, 2003; Gardner et al., 2005; Cane et al., 2014), dendritic morphogenesis, and spinogenesis (El-Husseini et al., 2000; Penzes et al., 2001; Hoogenraad et al., 2005; Nakamura et al., 2011; Geiger et al., 2014; Heisler et al., 2014), suggesting fundamental roles of these PDZ proteins in the development of the brain. However, although more than 400 PDZ proteins have been found so far, only a small part is studied and most of their functions remain elusive.

Among these PDZ proteins, the ligand of Numb protein X 1 (Lnx1) functions as an E3 ubiquitin ligase to promote proteasome-dependent degradation of Numb and enhance Notch signaling that has been revealed in cultured cell systems (Dho et al., 1998; Nie et al., 2002). We have revealed that Lnx1 is expressed in postsynaptic densities (PSDs) of hippocampal CA3 neurons and is required for mossy fiber (MF) axon targeting during the postnatal period (Liu et al., 2018). However, the postsynaptic function of Lnx1 remains largely unknown. In the present study, we further demonstrate that Lnx1 prevents EphB2 receptor internalization for the postsynaptic maturation. Constitutively active EphB2 receptor kinase in *Lnx*1^−/−^ mice is sufficient to rescue the postsynaptic structure and promote functional synapse formation. Thus, our data indicate that postsynaptic Lnx1-EphB complex is essential for hippocampal CA3 postsynaptic structure assembly and associated synaptic function in the developing brain.

## Materials and Methods

### Mice and Sample Preparation

*Lnx*1^−/−^ (Liu et al., 2018) knockout and *EphB*2^F620D^ (Holmberg et al., 2006) knockin mice and genotyping methods have been described previously. *Lnx*1^−/−^ mice were crossed with the Thy1-GFP M (Feng et al., 2000) transgenic mouse line, which were generously provided by Mark Henkemeyer (University of Texas Southwestern Medical Center, Dallas, TX, USA). Mice were anesthetized with isoflurane and perfused with 0.1 M phosphate-buffered saline (PBS) followed by 4% paraformaldehyde in phosphate buffer. The brains were then removed, postfixed, and sectioned at 30 μm using a vibratome. All experiments that involved mice were carried out in accordance with the US National Institutes of Health Guide for the Care and Use of Animals under an Institutional Animal Care and Use Committee approved protocol and Association for Assessment and Accreditation of Laboratory Animal Care approved Facility at the Shanghai Jiao Tong University School of Medicine. Mice were raised in animal facilities with a constant temperature (22°C) and on a 12-h light-dark cycle. Food and water were unlimited to access.

### Immunofluorescence

For immunofluorescence, vibratome sections were blocked with permeable buffer (0.3% Triton X-100 in PBS) that contained 10% donkey serum for 30 min at room temperature, incubated with primary antibodies in a permeable buffer that contained 2% donkey serum overnight at 4°C. The slices were then washed three times with PBS-T (0.1% Tween-20 in PBS) for 10 min every time and incubated with Alexa Fluor secondary antibodies (1:200, Molecular Probes) in the PBS buffer for 2 h at room temperature. The slices were washed in PBS-T three times and mounted on glass slides using Aqua-Poly/Mount (Polysciences) and photographed using a confocal microscope (Leica SP8). For primary antibodies, we used goat anti-EphB2 (1:500, R&D, P54763), rabbit anti-synapsin1 (1:1,000, gift from Ilya Bezprozvanny, University of Texas Southwestern Medical Center, Dallas, TX, USA), and rabbit anti-proteasome S20 (1:1,000, Abcam, ab3325).

To quantify the shape of the spine, a procedure was adapted from our previous study (Xu et al., 2011). To classify the shape of neuronal spines in slices, we used the NeuronStudio software package and an algorithm with the following cutoff values: aspect ratio (AR)_thin (crit) = 2.5, head to neck ratio (HNR) (crit) = 1.3, head diameter (HD) critical value (crit) = 0.4 μm. The protrusions with length 0.2–3.0 μm and Max width 3 μm were counted. Spine density was calculated by dividing the total spine number by the dendritic branch length. Primary dendrite was defined as the dendrite from the soma. The localizations of the terminals and spines were carefully identified in the 3D rendering views. The synapsin1^+^ spines were defined as the overlapped connections of spines and synapsin1^+^ terminals with any identical red/green pixels, otherwise synapsin1^−^ (separated from terminals). The neurons of the CA3 corner area were chosen and spines in the stratum oriens layer of the CA3 area were counted. Control and experiment conditions were adjusted with the same parameters. Acquisition of the images and morphometric quantification were performed under “blinded” conditions.

### Transmission Electron Microscopy

Mice were perfused with 2.5% glutaraldehyde in phosphate buffer, pH 7.2 for 30 min, dissected brains were then postfixed in the same buffer overnight at 4°C. After PBS buffer rinse, samples were postfixed in 1% osmium tetroxide buffer (2 h) on ice in the dark. Following a double-distilled water rinse, tissue was stained with 3% aqueous uranyl acetate (0.22 μm filtered, 1 h dark), dehydrated in a graded series of ethanol, propylene oxide, and embedded in Epoxy 618 resin. Samples were polymerized at 60°C for 48 h. Thin sections, 60–90 nm, were cut with a diamond knife on the LKBV ultramicrotome and picked up with Formvar-coated copper slot grids. Grids were stained with lead citrate and observed with transmission microscopy (PHILIP CM-120). Images from the CA3 corner area were chosen and an average of 8 pictures per mouse (*n* = 4 animals per genotype) were captured. The PSDs or PSD length was counted or analyzed by Image J software under “blinded” conditions.

### Primary Cell Culture

Primary cell culture of hippocampal neurons was performed as described (Xu and Henkemeyer, 2009). Briefly, hippocampal neurons were dissociated from postnatal day 0 (P0) pups. The triturated cells (1 × 10^5^ cells per well) were grown on either 6 well dishes or glass coverslips coated with 10 μM polylysine overnight in 24 well dishes. Then the culture was grown in a medium of Neurobasal A media (GIBCO) supplemented with B27 and 2 mM glutamine for indicated days.

### Receptor Trafficking and Internalization Assay

For receptor trafficking assay, cultured neurons were incubated with special N-terminus EphB2 antibodies (1:100, R&D, P54763) for 10 min. After three washes with preheated PBS, Alexa 488-conjugated secondary antibody (1:100) in blocking buffer was then applied for 10 min to label the total membranous receptors. After a rapid rinse with culture medium, neurons were placed back in the incubator with the original culture medium for 30 min to allow the trafficking of receptors. Then EphB2 antibodies (1:100) followed by Alexa 555-conjugated secondary antibody were applied for 10 min to label the trafficking receptors. After three washes with PBS, the neurons were fixed in 4% paraformaldehyde for 10 min and mounted.

For receptor internalization assay, cultured neurons were incubated with special N-terminus EphB2 antibodies (1:100, R&D, P54763) for 10 min. After a rapid rinse with a culture medium, neurons were placed back in the incubator for 30 min to allow the internalization of receptors. Alexa 488-conjugated secondary antibody (1:100) in blocking buffer was applied for 10 min to label the existing surface EphB2 receptors. After three washes with preheated PBS, the neurons were fixed in 4% paraformaldehyde for 10 min and permeabilized with a blocking buffer that contained 0.1% Triton-X 100 for 30 min. Alexa 555-conjugated secondary antibodies were then applied for 10 min to label the internalized population of receptors. Finally, the neurons were further incubated with proteasome S20 antibody (1:1,000, Abcam, ab3325) in a permeable buffer that contained 2% donkey serum overnight at 4°C and corresponding Alexa 647-conjugated secondary antibody (1:1,000) on the next day. After three washes with PBS, the neurons were mounted.

### Isolation of Cell-Surface Protein and Western Blotting

Primary hippocampal neurons from wild-type (WT) or *Lnx*1^−/–^ pups were cultured for 3 days, then GFP or FLAG-Lnx1 virus (OBIO Company, Shanghai, China) was added into the neurons for another 7-day culture. Then the primary hippocampal neurons were prepared for cell surface protein isolation and western blotting. Cell surface protein isolation of the cultured primary hippocampal neuron samples was separated using a Pierce Cell Surface Protein Isolation Kit (Thermo) as the manufacturer's protocol. Total and membrane proteins were separated by sodium dodecyl sulfate–polyacrylamide gel electrophoresis (SDS-PAGE), transferred to nitrocellulose membranes, and then immunoblotted with indicated antibodies. Analysis of the data was performed using NIH ImageJ software, the mean density of each band was normalized to β-actin signal in the same sample and averaged. For primary antibodies, we used mouse anti-β-actin (1:3,000, Thermo, MA5-15739), mouse anti-Flag (1:1.000, Sigma, F1804), and Goat anti-EphB2 (1:1,000, R&D, P54763).

### Electrophysiology

Brain coronal slices were prepared from 3-week-old naive *Lnx*1^+/+^, *Lnx*1^−/–^, *EphB*2^F620D^, and *Lnx*1^−/–^; *EphB*2^F620D^ mice. Brains were dissected quickly and chilled in ice-cold artificial cerebrospinal fluid (ACSF) that contains (in mM): 125 NaCl, 2.5 KCl, 2 CaCl_2_, 1 MgCl_2_, 25 NaHCO_3_, 1.25 NaH_2_PO_4_, and 12.5 Glucose. Coronal brain slices (300 μm thick) were prepared with a vibratome and recovered in ACSF bubbling with 95% O_2_ and 5% CO_2_ at 31°C for 1 h and then maintained at room temperature (22–25°C). The electrode internal solution was composed of 115 mM CsMeSO_3_, 10 mM HEPES, 2.5 mM MgCl_2_·6H_2_O, 20 mM CsCl_2_, 0.6 mM ethylene glycol tetraacetic acid (EGTA), 10 mM Na_2_phosphocreatine, 0.4 mM Na-guanosine 5′-triphosphate (GTP), and 4 mM Mg-adenosine triphosphate (ATP). For mEPSC recording, tetrodotoxin (1 μM) and picrotoxin (100 μM) were included in the external solution. Cells were held at −70 mV. Miniature responses were acquired with a Multiclamp 200B at 10 kHz. Prior to mEPSC detection and analysis, current traces were low-pass filtered at 5 kHz. Data were analyzed in pClamp 10.6 (Molecular Devices) and recordings were made from an average of 3 cells per slice and 2–3 slices per mouse.

### Statistical Analysis

The results are presented as mean ± SEM. Statistical differences were determined by Student's t-test for two-group comparisons or ANOVA followed by Turkey test for multiple comparisons among more than two groups.

## Results

### The Ligand of Numb Protein X 1 Is Required for CA3 Spinogenesis and Synapse Formation

**Figure 1:**
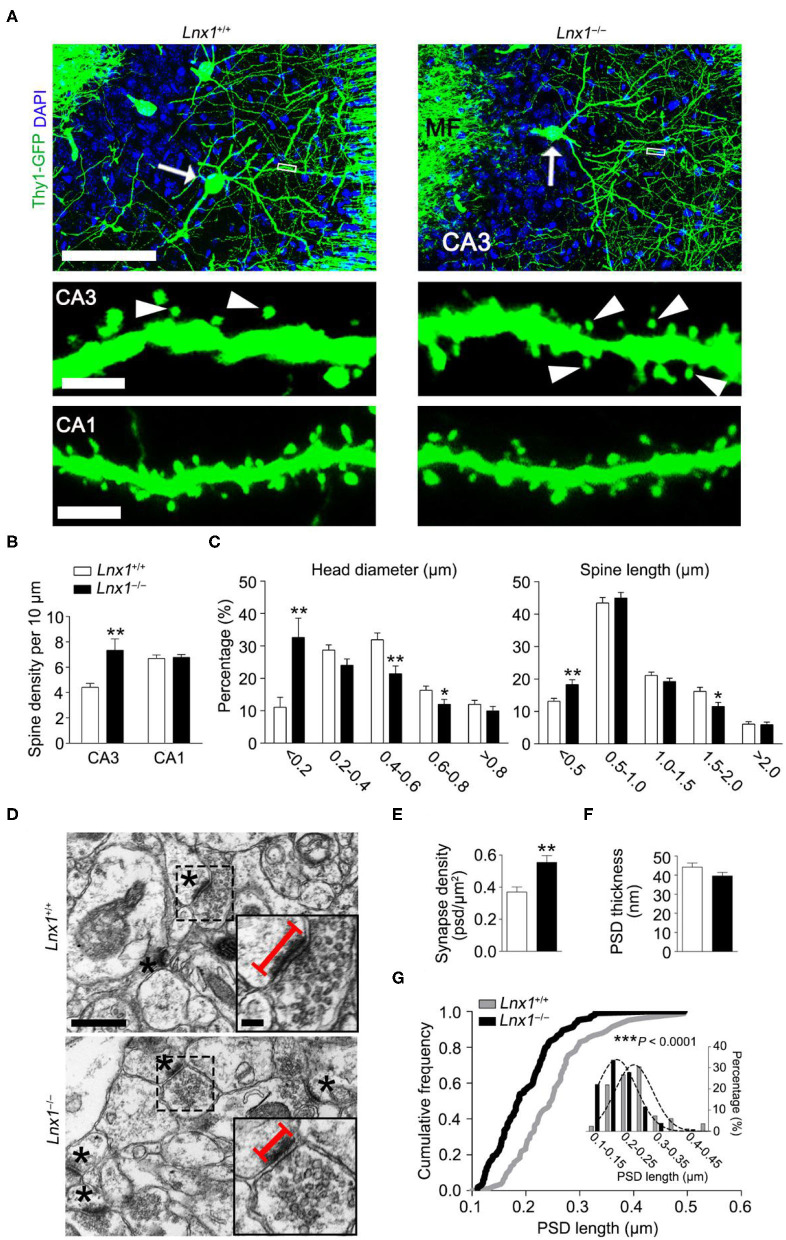
Ligand of Numb protein X 1 (Lnx1) is required for spinogenesis in CA3 neuron. **(A)** Spine morphogenesis in CA3 and CA1 neurons from PW3 *Lnx*1^+/+^ and *Lnx*1^−/−^ mice. Arrows delineate neurons (upper panel) with the mushroom spine (arrowheads in lower panel). Scale bars represent 100 μm (upper panel), 2.5 μm (middle panel), and 3 μm (lower panel). MF, Mossy Fiber. **(B,C)** Quantification of the spine density in CA3 neurons and CA1 neurons. Percentage distribution of spine head diameter and spine length in CA3 neurons. *n* = 39 neurons from 4 mice in wild-type (WT) and 43 neurons from 5 mice in the *Lnx*1^−/−^ group. **(D)** Transmission electron micrographs of CA3 regions. Asterisks mark postsynaptic spines. Straight lines denote postsynaptic density (PSD) length. Scale bars represent 0.5 μm (left panel) and 0.1 μm (right panel). **(E)** Quantification of synapse density in the CA3 region determined from electron microscopy analysis. *n* = 37 images from 4 mice in WT and 42 images from 4 mice in the *Lnx*1^−/−^ group. **(F,G)** Quantification of PSD thickness and PSD length in the CA3 region determined from electron microscopy analysis. The right panel presents a cumulative frequency plot of PSD length with histogram distribution and Gaussian curve fit for the inset. *n* = 82 synapses from 4 mice in WT and 104 synapses from 4 mice in the *Lnx*1^−/−^ group. Data are presented as mean ± SEM. *p < 0.05; **p < 0.01; ***p < 0.001.

We have identified that Lnx1 was expressed especially in the postsynaptic fraction of hippocampal CA3 neurons (Liu et al., 2018), the postsynaptic localization prompted us to detect whether neuronal morphogenesis is altered in *Lnx*1^−/−^ mice. We compared the dendrites and spines between postnatal week 3 (PW3) *Lnx*1^−/−^ and WT mice with a Thy1-GFP-M transgenic reporter (Feng et al., 2000) and found a higher spine density of CA3 but not CA1 pyramidal neurons in *Lnx*1^−/−^ mice than that of WT littermate mice (Figures 1A,B), though no difference was observed in primary dendrites (data not show). Analysis of spine morphology showed that the spine head width and spine length in CA3 pyramidal neurons of *Lnx*1^−/−^ mice were significantly decreased (Figures 1A,C). These results indicated that the deficiency of lnx1 in CA3 neurons may lead to changes in the morphology and function of dendritic spines. To examine whether the changes in spine density and morphology resulted in changes in synapse number or postsynaptic structure, we further performed transmission EM and analyzed the number of synapses in the CA3 area and their postsynaptic morphology in PW3 mice. We observed a significant increase in the density of synapses with a decreased postsynaptic profile area indicated by its PSD length in *Lnx*1^−/−^ mice as compared to WT mice (Figures 1D–G). These results demonstrate a critical role of Lnx1 in the process of spinogenesis in adolescent mice.

**Figure 2:**
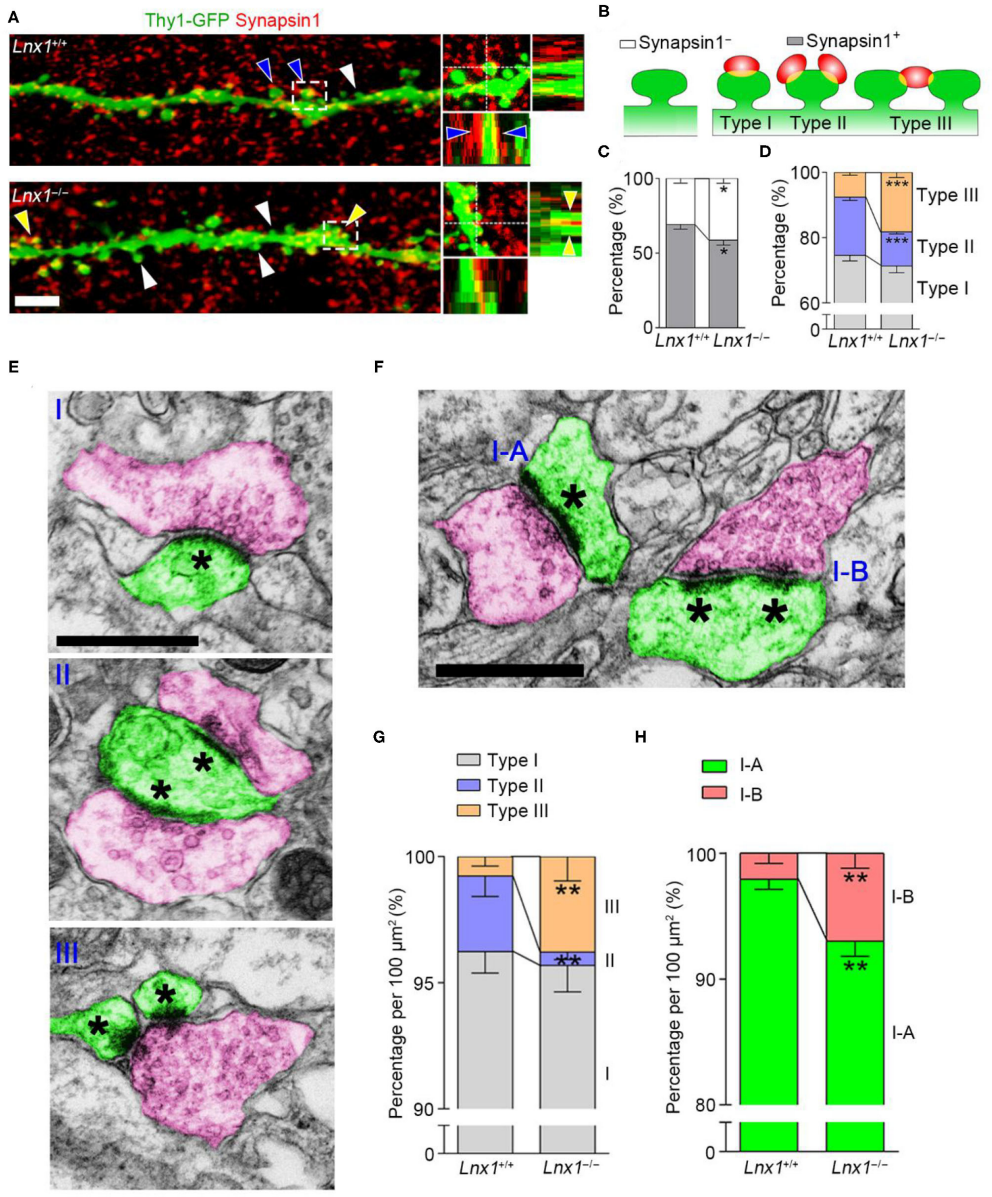
Ligand of Numb protein X 1 (Lnx1) is required for synapse formation in CA3 neuron. **(A)** Representative images showing the deconvolved 3D-rendered views of spines of CA3 neuron (green) contacting synapsin1 (red) in PW3 WT and *Lnx1*^−/−^ mice (left). Cross-sections through the x- and y-axes show the interface between the spines and synapsin1^+^ terminals (right). White arrowheads indicate synapsin1^−^ spine, blue arrowheads indicate type II synapse, yellow arrowheads indicate type III synapse. Scale bar represents 4 μm. **(B)** Schematic diagram showing synapsin1^−^ spine and synapsin1^+^ spines which include three types of synapse. **(C,D)** Percentage of various synapse types determined from 3D-rendered views in WT and *Lnx*1^−/−^ mice. *n* = 14 neurons from 4 mice in WT and 16 neurons from 4 mice in the *Lnx1*^−/−^ group. **(E,F)** Schematic of various synapse types that include presynaptic terminals (red) and postsynaptic spines (green) in electron microscopy. Asterisks mark postsynaptic density (PSD). Scale bar represents 0.5 μm. **(G,H)** Percentage of various synapse types analyzed by electron microscopy from WT and *Lnx*1^−/−^ mice. *n* = 592 synapses from 4 mice in WT and 475 synapses from 4 mice in the *Lnx*1^−/–^ group. Data are presented as mean ± SEM. *p < 0.05; **p < 0.01; ***p < 0.001.

The abnormal spine morphogenesis in *Lnx*1^−/−^ mice promotes us to ask if the structure of synapses is anatomically altered. Brain slices from PW3 WT and *Lnx*1^−/−^ mice with Thy1-GFP-M transgenic reporter were immunostained with anti-synapsin1, a protein marker for presynaptic terminals. In WT mice, most spines of CA3 neurons were contacted with presynaptic terminals (synapsin1^+^ spine), while this ratio was decreased in *Lnx*1^−/−^ mice (Figures 2A–C). We further classified the synapsin1^+^ spines of CA3 neurons into three types based on their morphology: one presynaptic terminal on one spine (Type I, typical 1:1 spine), two or more presynaptic terminals on one spine (Type II, multi-innervated spines), and one presynaptic terminal on two or more spines (Type III, multi-spine boutons) (Figures 2A,B). We observed a differential distribution of the three type synapses, Type I the highest and Type III the lowest, in WT mice. However, *Lnx*1^−/−^ mice showed an increased rate of Type III synapse and a decreased rate of Type II synapse (Figure 2D). To validate the phenotype observed by immunofluorescence staining, we performed transmission EM to analyze these synapse types (Figure 2E). We observed an increased rate of Type III synapse and a decreased rate of Type II synapse, while the percent of Type I synapse remains unchanged in *Lnx*1^−/−^ mice when compared with littermate controls (Figure 2G). We further classified the Type I synapse into two subtypes, continuous PSDs (Type I-A) and segmented PSDs (Type I-B) (Figure 2F), and observed a significantly increased ratio of Type I-B to Type I-A synapse in *Lnx*1^−/−^ mice (Figure 2H). Combined with our previous results that postsynaptic efficacy was greatly reduced in the CA3 neurons of *Lnx*1^−/−^ mice (Liu et al., 2021), these results thus demonstrate a critical role for Lnx1 to establish an intact postsynaptic structure that attributes to the formation of functional synapses in adolescence mice.

### The Ligand of Numb Protein X 1 Participates in the Internalization Trafficking of EphB Receptors

**Figure 3:**
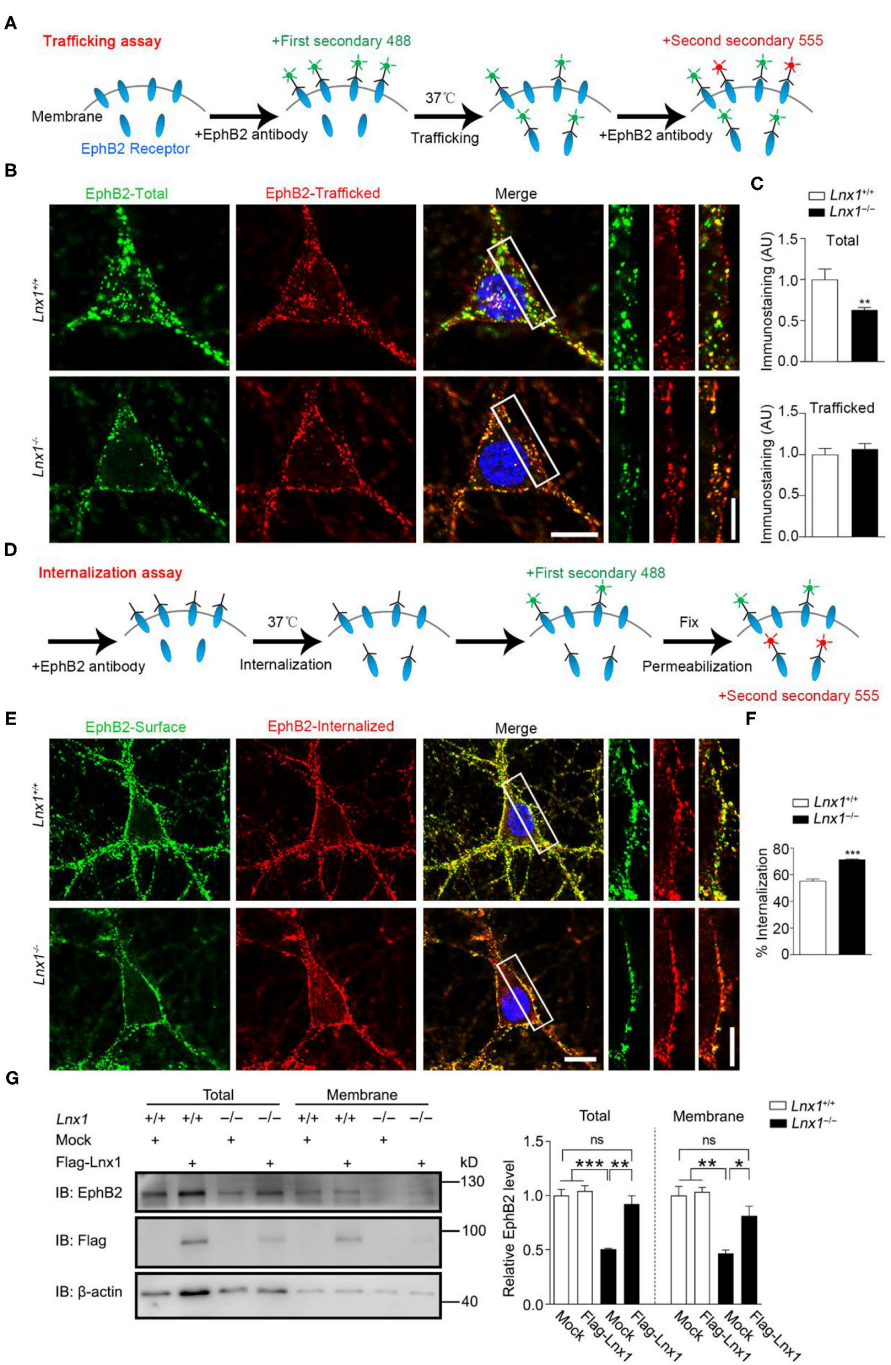
Ligand of Numb protein X 1 (Lnx1) knockout affects the internalization of EphB2 receptors. **(A)** Schematic diagram shows the strategy to label the trafficking of EphB2 receptors. **(B)** Trafficking of EphB2 receptors in *Lnx*1^+/+^ and *Lnx*1^−/−^ hippocampal neurons. Green, total membranous EphB2 receptors; red, newly trafficked EphB2 receptors. Scale bars represent 10 μm (left) and 5 μm (right). **(C)** Statistical analysis of total and trafficked EphB2 receptors. *n* = 20 neurons from three biological replicates in wild-type (WT) and 19 neurons from three biological replicates in the *Lnx1*^−/−^ group. **(D)** Schematic diagram shows the strategy to label the internalization of EphB2 receptors. **(E)** Internalization of EphB2 receptors in *Lnx*1^+/+^ and *Lnx*1^−/−^ hippocampal neurons. Green, surface EphB2 receptors; red, internalized EphB2 receptors. Scale bars represent 10 μm (left) and 5 μm (right). **(F)** Statistical analysis of the internalized ratio of EphB2 receptors. *n* = 22 neurons from three biological replicates in WT and 21 neurons from three biological replicates in the *Lnx1*^−/−^ group. **(G)** Total level or membrane level of EphB2 receptor were analyzed by western blotting of primary hippocampal neurons from WT or *Lnx1*^−/−^pups with or without overexpression of Lnx1 protein. ns indicates no significant. Quantitative results of three biological replicates are shown. Data are presented as mean ± SEM. *p < 0.05; **p < 0.01; ***p < 0.001.

In the previous study, we screened molecules that were involved in synaptogenesis mediated by Lnx1 and found that the EphB receptors, a family of tyrosine receptor kinases that regulate excitatory synaptogenesis early during development (Sheffler-Collins and Dalva, 2012), were reduced in cell membrane component extracted from *Lnx*1^−/−^ hippocampus. Lnx1 stabilized EphB receptors through specific binding sites to prevent their degradation in the proteasome (Liu et al., 2018). Lnx1 knockout decreased the surface localization of EphB receptors, which could be the result of either impaired trafficking of EphBs receptors into the plasma membrane or enhanced internalization of EphBs from the plasma membrane. To further determine how Lnx1 regulated the surface expression of EphB receptors, we cultured *Lnx*1^−/−^ hippocampal neurons and investigated the surface trafficking of the EphB2 receptor. We firstly used a special antibody against the N-terminus of the EphB2 receptor and corresponding fluorescence secondary antibody (488, green) to label the already existing surface EphB2 receptors at DIV10 (day *in vitro*), after a 30 min trafficking, we used N-terminal EphB2 antibody again and another corresponding fluorescence secondary antibody (555, red) to label new surface EphB2 receptors that trafficked from cytoplasm to membrane in the 30-min period (Figure 3A). We observed the surface EphB2 receptors (488, green) were decreased in the *Lnx*1^−/−^ hippocampal neurons when compared with WT neurons, which was inconsistent with our previous results that loss of Lnx1 reduced surface EphB receptors (Figures 3B,C). However, the newly trafficked EphB2 receptors (555, red) showed no difference between WT and *Lnx*1^−/−^ neurons (Figures 3B,C), indicating that Lnx1 did not affect the trafficking of EphB receptors into the plasma membrane.

Then we investigated whether the internalization of EphB2 receptors from the plasma membrane was affected by Lnx1. Similarly, we used an antibody against the N-terminus of the EphB2 receptor to bind the surface EphB2 receptors and allow them to internalize from the plasma membrane. We added the corresponding fluorescence secondary antibody (488, green) to label the existing surface EphB2 receptors after 30-min incubation, then neurons were fixed and permeabilized for another corresponding fluorescence secondary antibody (555, red) to label the internalized EphB2 receptors from the plasma membrane (Figure 3D). We found that the ratio of internalized EphB2 was increased in *Lnx*1^−/−^ hippocampal neurons as compared to WT neurons (Figures 3E,F). Furthermore, we extracted the membrane fraction of cultured primary hippocampal neurons from WT or *Lnx*1^−/−^ pups and observed a decreased EphB2 level in both total and membrane fractions in *Lnx*1^−/−^ neurons, which could be restored to a comparable normal level after the overexpression of Lnx1 protein (Figure 3G). Together, these data suggest that Lnx1 is necessary and sufficient for the stability of EphB receptors by preventing their internalization from the cell surface.

### Activating EphB2 Kinase Promotes Synapse Maturation in *Lnx*1^–/–^ Mice

**Figure 4:**
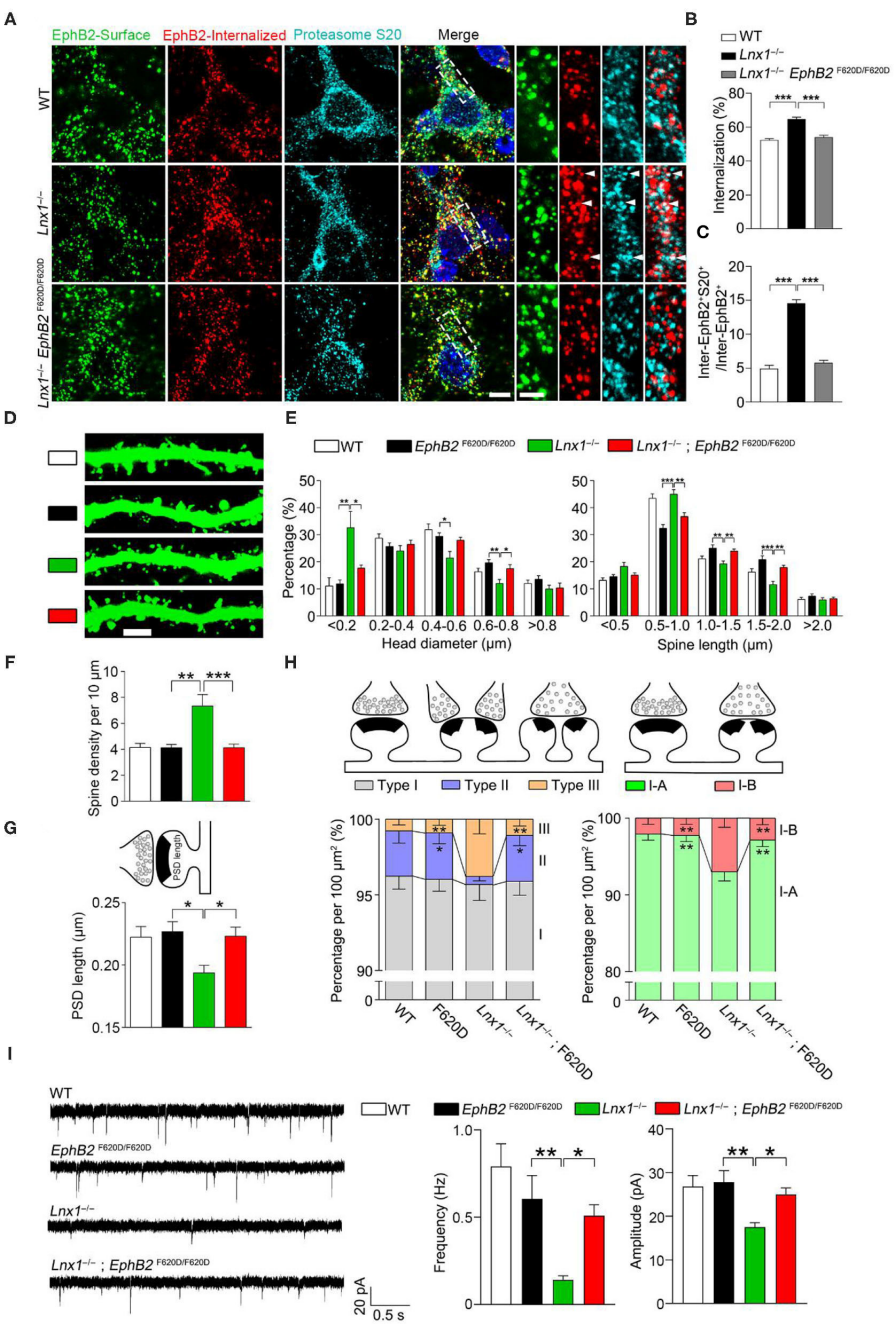
EphB kinase activation promotes synaptogenesis. **(A)** Internalization of EphB2 receptors and immunofluorescent staining with anti-proteasome S20 in wild-type (WT), *Lnx*1^−/−^, and *Lnx*1^−/−^*; EphB2* ^F620D/F620D^ hippocampal neurons. Green, surface EphB2 receptors; red, internalized EphB2 receptors; cyan, proteasome S20. White arrowheads indicate co-localization of internalized EphB2 and proteasome S20. Scale bars represent 5 μm (left) and 2 μm (right). **(B)** Statistical analysis of the internalized ratio of EphB2 receptors. **(C)** Quantification of internalized EphB2 and S20 co-localization (inter-EphB2^+^S20^+^) / internalized EphB2 (Inter-EphB2^+^). *n* = 20 neurons from three biological replicates in WT, 20 neurons from three biological replicates in *Lnx*1^−/−^ and 21 neurons from three biological replicates in *Lnx*1^−/−^*; EphB2* ^F620D/F620D^. **(D**,**E)** Spine morphogenesis and the quantification of percentage distribution of spine head diameter and spine length in CA3 neurons from PW3 WT, *EphB2* ^F620D/F620D^, *Lnx*1^−/−^, and *Lnx*1^−/−^*; EphB2* ^F620D/F620D^ mice. Scale bars represent 3 μm. **(F)** The increased spine density in *Lnx*1^−/−^ mice was restored to normal level in *Lnx*1^−/−^; *EphB2* ^F620D/F620D^ mice. *n* = 39 neurons from 4 mice in WT, 27 neurons from 3 mice in *EphB2* ^F620D/F620D^, 43 neurons from 5 mice in the *Lnx*1^−/–^ group, and 36 neurons from 4 mice in *Lnx*1^−/–^; *EphB2* ^F620D/F620D^ group. **(G)** The shorter PSD length in *Lnx*1^−/−^ mice was restored to normal level in *Lnx*1^−/−^; *EphB2* ^F620D/F620D^ mice. *n* = 80 synapses in WT, 93 synapses in *Lnx*1^−/–^, 72 synapses in *EphB2* ^F620D/F620D^, and 88 synapses in *Lnx*1^−/−^*; EphB2* ^F620D/F620D^ from 4 mice per group. **(H)** Schematic and quantification of different synapse types in WT, *EphB2* ^F620D/F620D^, *Lnx*1^−/−^, and *Lnx*1^−/−^*; EphB2* ^F620D/F620D^ mice determined from electron microscopy analysis. *n* = 592 synapses in WT, 475 synapses in *Lnx*1^−/–^, 393 synapses in *EphB2* ^F620D/F620D^, and 534 synapses in *Lnx*1^−/−^*; EphB2* ^F620D/F620D^ from 4 mice per group. **(I)** Representative average traces (left) and summary graph (right) showed that decreased mEPSC frequency and amplitude in PW3 *Lnx*1^−/−^ mice were restored to normal level in *Lnx*1^−/−^*; EphB2* ^F620D/F620D^ mice. *n* = 15 neurons from 3 mice in WT, 18 neurons from 4 mice in *EphB2* ^F620D/F620D^, 17 neurons from 4 mice in the *Lnx*1^−/–^ group, and 20 neurons from 4 mice in *Lnx*1^−/–^; *EphB2* ^F620D/F620D^ group. Data are presented as mean ± SEM. *p < 0.05; **p < 0.01; ***p < 0.001.

We have found that Lnx1 stabilized EphB receptors by preventing their degradation in proteasome in our previous study (Liu et al., 2018). To test whether the internalized EphB2 receptors in *Lnx*1^−/−^ hippocampal neurons were subsequently submitted to proteasome for degradation, we performed internalization assays and immunofluorescence staining with anti-proteasome S20 to label the proteasomes. In WT neurons, we observed little co-localization between EphB2 receptors and proteasomes, while a portion of internalized EphB2 receptors in *Lnx*1^−/−^ neurons were co-localized with proteasome S20 protein (Figures 4A–C), suggesting that Lnx1 is necessary for arresting EphB2 receptor internalization for proteasomal degradation.

**Figure 5:**
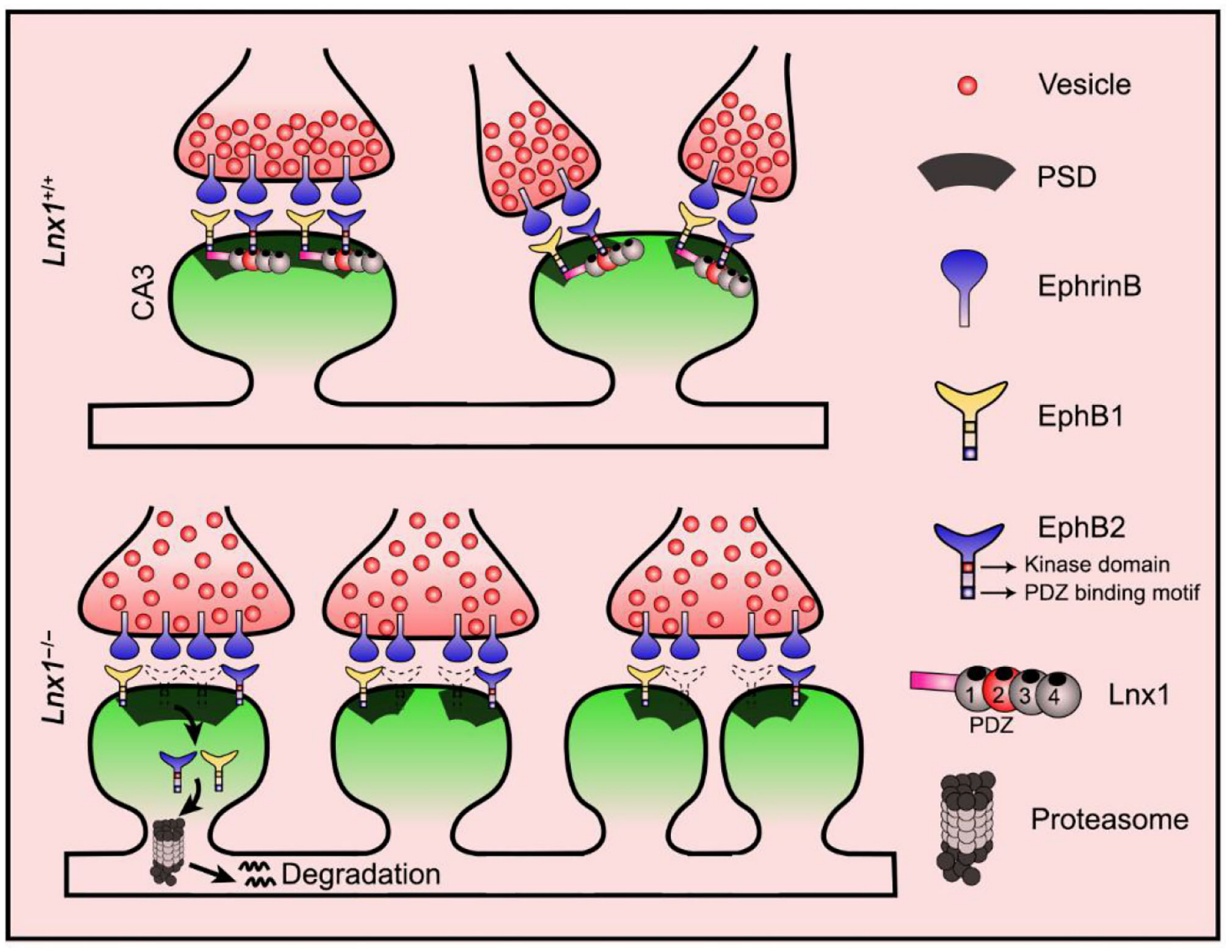
Proposed model for diversiform synaptogenesis through Lnx1-EphB signaling. Ligand of Numb protein X 1 (Lnx1) serves as a specific protein stabilizer for postsynaptic EphB receptors in hippocampal CA3 pyramidal neurons to sculpt postsynaptic structure and promote diversiform synaptogenesis during the developing hippocampus. PSD, postsynaptic density.

EphB2 forward signaling has been reported to be required for the spine morphogenesis and synapse formation in the CA3 pyramidal neurons *in vivo* (Henkemeyer et al., 2003). To determine whether constitutive catalytic activation of EphB2 is able to reverse the morphological defect caused by Lnx1 ablation, *EphB*2^F620D/F620D^, a constitutively active form of EphB tyrosine kinase (Holmberg et al., 2006), was crossed with *Lnx*1^−/−^ mice. We first detected the ratio of internalized EphB2 receptors in WT, *Lnx*1^−/−^ and *Lnx*1^−/−^; *EphB*2^F620D/F620D^ primary hippocampal neurons, and found that the increased ratio of internalized EphB2 in *Lnx*1^−/−^ was recovered in *Lnx1* ^−/−^; *EphB*2^F620D/F620D^ neurons (Figures 4A,B). Moreover, the ratio of co-localization between internalized EphB2 receptors and proteasomes was also restored to a comparable normal level in *Lnx*1^−/−^; *EphB*2^F620D/F620D^ neurons (Figure 4C), which suggest that active EphB2 is more resistant to the degradation. We next examined the spine morphology of CA3 neurons and found that the *EphB*2^F620D/F620D^ mice *per se* remain unchanged when compared to the WT mice, while the shrunken spines in CA3 neurons were completely recovered in *Lnx*1^−/−^; *EphB*2^F620D/F620D^ compound mutants (Figures 4D–F). We also examined the rearrangement of all types of synapses and found that the *EphB*2^F620D/F620D^ mice *per se* showed no difference when compared to WT mice in synapse type percentage (Figure 4G). The decreased Type II and increased Type III and upregulated I-B/I-A ratio were significantly restored in *Lnx*1^−/−^; *EphB*2^F620D/F620D^ compound mutants compared with *Lnx*1^−/−^ mice (Figure 4H). Finally, to determine whether constitutive catalytic activation of EphB2 can rescue synaptic efficacy in *Lnx*1^−/−^ neurons, we measured synaptic activity in CA3 pyramidal neurons from acute brain slices of PW3 mice. Both frequency and amplitude of mEPSC were recovered to WT level in *Lnx*1^−/−^; *EphB*2^F620D/F620D^ compound mutants (Figure 4I). Taken together, our data are consistent with a model whereby Lnx1 serves as a specific protein stabilizer for postsynaptic EphB receptors in hippocampal CA3 pyramidal neurons to promote diversiform spinogenesis and synaptic remodeling (Figure 5).

## Discussion

In this study, we reveal PDZ scaffold protein Lnx1, which serves as a membrane stabilizer to sustain receptor proteins at postsynaptic compartments to control the formation of diversiform synapses in the developing hippocampus. In contrast to our previous study that Lnx1 controls the axon targeting and maturation of MF terminals in a non-cell-autonomous manner (Liu et al., 2018), we reveal that Lnx1 functions as an intrinsic determinant of postsynaptic structure maturation and synapse function by sustaining EphB2 receptor proteins at the cell surface to activate forward kinase signaling.

The postsynaptic structure has long been theorized to be a component of memory, which is enhanced with increased synapse density in the hippocampus (Moser, 1999; Geinisman, 2000; Leuner and Shors, 2004; Segal, 2005). On the contrary, through a genetic targeting approach, we found that the increased synapse density of CA3 pyramidal neurons during hippocampal development, caused by Lnx1 deficiency, results in an impaired social memory as shown in our previous study (Liu et al., 2021). Here, we suggest that this might be attributable to the numerous immature spines in *Lnx*1^−/−^ mice, that this is either unable to connect with presynaptic terminals as shown in Figures 2A–C or merely forms silent synapses with weak synaptic efficacy, which has been validated in our previous studies (Liu et al., 2018, 2021). These results indicate that the CA3 synapse is impaired in the absence of Lnx1 as the hippocampus develops, which may lead to damage to social memory.

In our study, we observed an increase in perforated (Type I-B) and multi-spine synapses (Type III) companied with a reduction in single synapses (Type I-A) in 3-wk-old *Lnx*1^−/−^ mice when compared to WT mice possessing a high proportion of single synapses but little perforated synapse or multi-spine synapses. Previous studies have shown that multiple spine synapses (Type III) and perforated synapses (Type I-B) showed a timely increase during the LTP induction, which may represent an intermediate state of synapses that probably transformed to mature and functional synapses eventually (Buchs and Muller, 1996; Toni et al., 1999). The vast majority of multiple spine synapses (Type III) had spines from different dendrites, while the number and ratio from the same dendrite were greatly increased during LTP induction (Toni et al., 1999). We thought that the increased multiple spine synapses (Type III) in *Lnx*1^−/−^ mice may be originated probably from the same dendrite that was similar to LTP stimulation. We also observed more multiple spine synapses (Type III) that have both spines on the same dendrite in *Lnx*1^−/−^ mice when compared to that in WT mice with immunofluorescence staining as shown in Figure 2A. Upon LTP induction, single synapse (Type I-A) becomes an unstable perforated synapse (Type I-B) through an unknown mechanism, and this results in the formation of multiple single synapses (Type I-A), eventually increasing the total number of synapses (Luscher et al., 2000; Harris et al., 2003), which may explain why more synapses were found in *Lnx*1^−/−^ mice. In addition, the Type II structures (spine with multiple excitatory contacts) formed by the competition between neighboring inputs for postsynaptic resources are found more frequently during the development than in adulthood (Sorra et al., 1998; Radwanska et al., 2011; Risher et al., 2014). Thus, our results may uncover a novel mechanism for dynamical modulation of structural changes in the formation of synapses during postnatal development.

In the present study, we find that Lnx1 is required for the stabilization of EphB2 on the cell surface. Loss of Lnx1 promotes the internalization and degradation of EphB2. We observed that a portion of the internalized EphB2 receptors in *Lnx*1^−/−^ neurons were co-localized with proteasome S20 proteins upon their internalization from the cell surface, thus, our data suggest a model in which EphB2 receptors were internalized quickly from the cell surface and subsequently sent to the proteasome for degradation in the absence of Lnx1. EphB receptors may be integrated differently in a heterogeneous molecular complex by Lnx1 to regulate variable synaptic structure dynamics. As the forward signals mediated by membrane EphB receptors in CA3 pyramidal neurons is essential for spinogenesis and postsynaptic structure remodeling (Henkemeyer et al., 2003), we crossed the EphB2 mutant with constitutively active forward signaling (*EphB*2^F620D/F620D^) mice with *Lnx*1^−/−^ mice to observe an obvious rescue in synapse formation. Mechanistically, secreted glycoprotein Reelin can bind to the extracellular domain of the EphB receptor to induce forward signaling, which works together with ApoER2 and VLDL receptor cascades to regulate neuron cytoskeleton and cellular behavior in CA3 neurons (Bouche et al., 2013). In particular, EphB receptor signaling in postsynaptic specializations can also be induced by the PDZ scaffold proteins PICK1 and GRIP1, to regulate the synapse formation and functions (Sheffler-Collins and Dalva, 2012; Sloniowski and Ethell, 2012). Thus, our study adds a new regulatory member in the large macromolecular complex.

Accumulating pieces of evidence have revealed the critical role of EphB receptors in brain development and function (Sheffler-Collins and Dalva, 2012; Klein and Kania, 2014; Kania and Klein, 2016) and genetic and protein linkage of EphB signal deficit in the hippocampus to Alzheimer's disease (Attwood et al., 2011), autism (Sanders et al., 2012), and Angelman syndrome (Margolis et al., 2010). Besides the EphB receptor, Lnx1 involved a protein interaction network of postsynaptic compartments (Wolting et al., 2011; Guo et al., 2012) and of alternatively spliced isoform that links genetic risk factors for autism spectrum disorders (Corominas et al., 2014). Therefore, our analysis clarifies the mechanisms by which structural and functional synapse assembly is regulated in the developing brain and leads to a greater understanding of the molecular basis for brain wiring and cognitive functions.

## Data Availability Statement

The raw data supporting the conclusions of this article will be made available by the authors, without undue reservation.

## Ethics Statement

The animal study was reviewed and approved by Animal Care and Use Committee. The Association for Assessment and Accreditation of Laboratory Animal Care approved the facility at the Shanghai Jiao Tong University School of Medicine.

## Author Contributions

NL performed the experiments of morphology and histology. SC and SS assisted with electrophysiology. N-JX and J-JC initiated the Lnx1 project. NL and X-DL designed experiments and wrote the manuscript. All authors contributed to the article and approved the submitted version.

## Funding

This research was supported by the National Natural Science Foundation of China (32030042 and 81870820 to N-JX, 81970997 to SS, and 31900796 to X-DL), the Science and Technology Commission of Shanghai Municipality (18JC1420302), and the Innovation Program of Shanghai Municipal Education Commission (2017-01-07-00-01-E00046).

## Conflict of Interest

The authors declare that the research was conducted in the absence of any commercial or financial relationships that could be construed as a potential conflict of interest.

## Publisher's Note

All claims expressed in this article are solely those of the authors and do not necessarily represent those of their affiliated organizations, or those of the publisher, the editors and the reviewers. Any product that may be evaluated in this article, or claim that may be made by its manufacturer, is not guaranteed or endorsed by the publisher.
